# Supplementary material for: Thermal and Calorimetric Investigations of Some Phosphorus-Modified Chain Growth Polymers 1: Polymethyl Methacrylate
Source: Polymers (Basel). 2022 Apr 1;14(7):1447. doi: 10.3390/polym14071447 (PMC9002683; doi:10.3390/polym14071447)
Supplement: Supplementary file 1 [file polymers-14-01447-s001.zip › polymers-1628574-supplementary.pdf]

polymers

MDPI

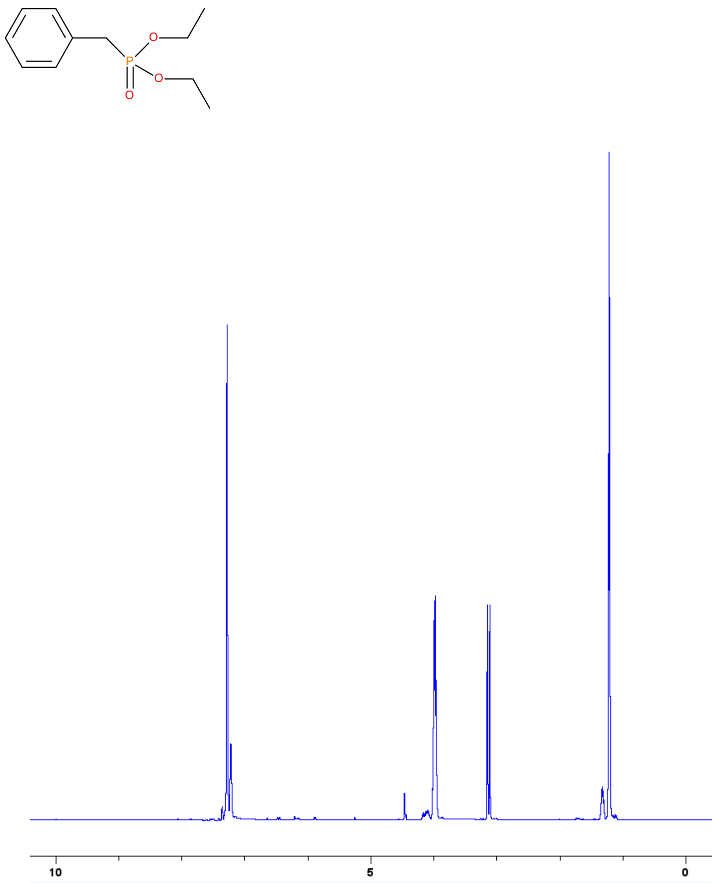

Figure S1. 600 MHz ^{1}H NMR spectrum of diethylbenzylphosphonate (J_{HP} = 21.9 Hz).

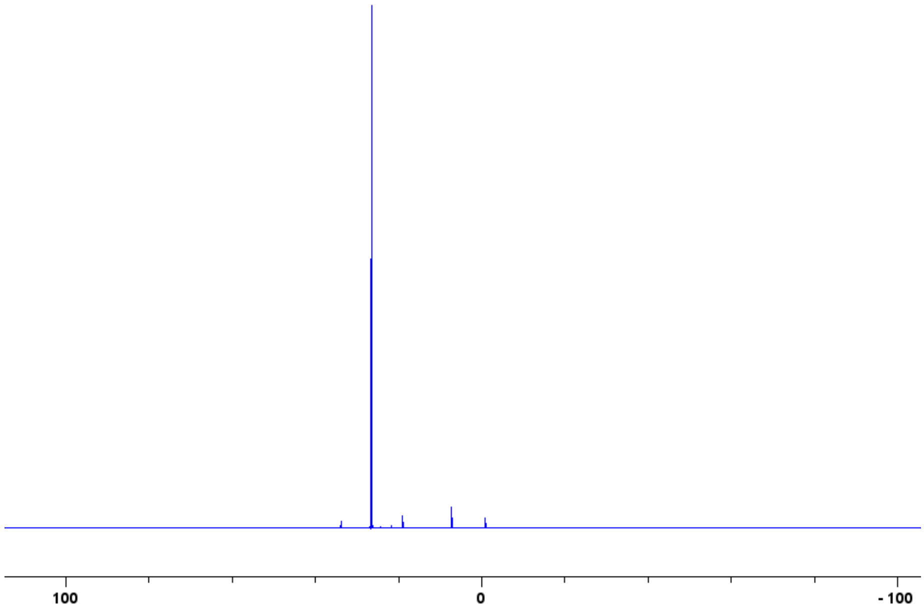

Figure S2. 600 MHz ^{31}P NMR spectrum (proton decoupled) of diethylbenzylphosphonate (∂ = 26.4 ppm).

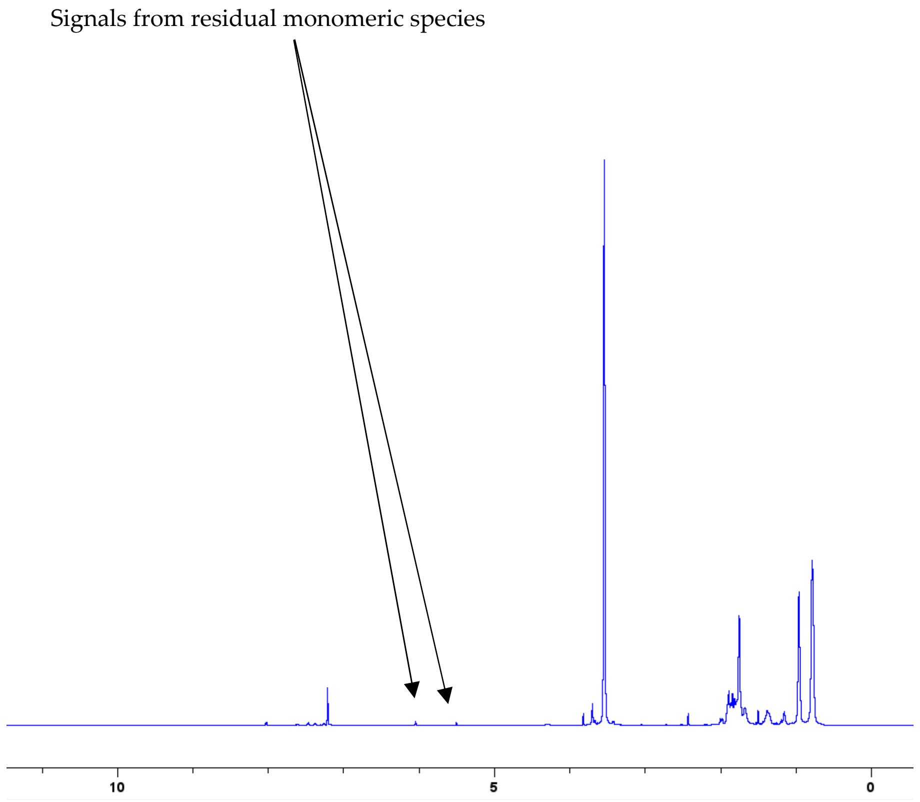

Figure S3. 600 MHz ^{1}H NMR spectrum of polymethyl methacrylate.

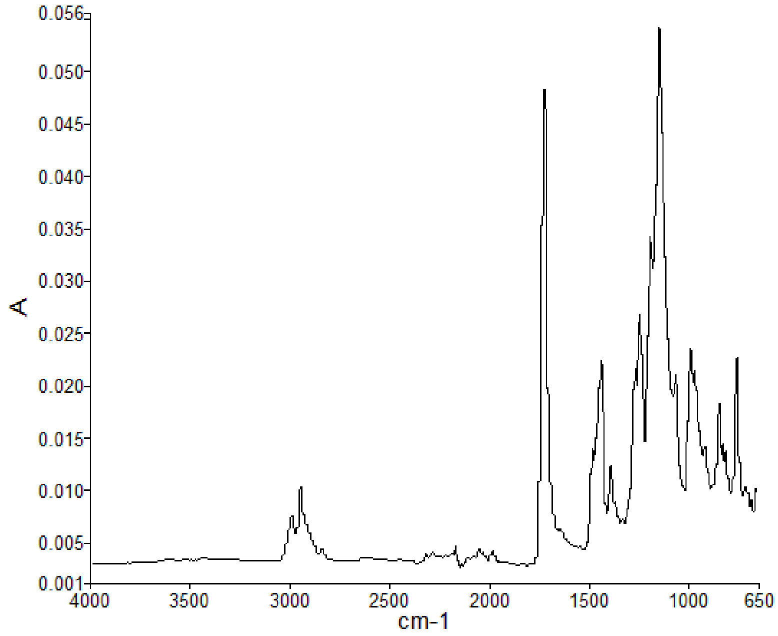

Figure S4. FT-IR spectrum of polymethyl methacrylate (neat: total attenuated reflectance mode)-note here the signals expected from –C=C- stretching (~1640 cm^{-1}) from the vinyl function of unspent monomeric species.
